# The complete mitochondrial genome of *Rhynchocypris czekanowskii* (Cypriniformes, Cyprinidae)

**DOI:** 10.1080/23802359.2025.2535639

**Published:** 2025-07-23

**Authors:** Yin-Tao Zhang, Cheng-Pu Lu, Yu-Hui Tao, Cheng-Wei Tong, Jie Chen, Wei Liu

**Affiliations:** a Zhejiang Lishui Service Platform for Technological Innovations in Traditional Chinese Medicine Industry (ZJLS-TCMTI SP), Lishui University, Lishui, China; b Qingyuan Bureau of Natural Resources and Planning, Lishui, China; c College of Ecology, Lishui University, Lishui, China; d Forestry Bureau of Jinyun County, Lishui, China; e Forestry Bureau of Lishui City, Lishui, China

**Keywords:** Phylogenetic analysis, Northeast Asian freshwater fish, genome structure, molecular conservation

## Abstract

Here we present the first complete mitochondrial genome assembly of *Rhynchocypris czekanowskii* Dybowski, 1869. We characterized a 16,651-bp circular genome comprising 13 protein-coding genes, 22 tRNA genes, 2 rRNA genes, and a 981-bp control region. Phylogenetic reconstruction using mitochondrial sequences robustly resolved *R. czekanowskii* as sister to *R. lagowskii* (bootstrap = 100%), supporting morphological affinities. This work fills a critical genomic gap in *Rhynchocypris* phylogenetics and has significant implications for both taxonomic revision and the conservation of cold-adapted ichthyofauna in Northeast Asia.

## 1. Introduction

*Rhynchocypris czekanowskii* Dybowski, 1869, a small-bodied cyprinid fish endemic to Northeast Asian cold-water ecosystems, serves as a keystone species across multiple river basins including the Yenisei, Heilongjiang, Yalu, and Xiaoling systems (Zuev et al. 2019). This taxon displays remarkable ecophenotypic adaptations, featuring an elongated silvery morphology (standard length 65–85 mm) with reduced lateral pigmentation and a distinctive mid-lateral melanic stripe—morphological specializations that reflect its ecological preference for clear lentic waters with dense macrophyte cover (Freyhof and Kottelat 2007).

Mitochondrial data are particularly valuable for phylogenetic and conservation studies due to the maternal inheritance of mitochondrial DNA and its relatively high mutation rate, which make it a useful tool for resolving evolutionary relationships and identifying cryptic biodiversity. Despite its biogeographic significance and ecological prominence, this species remains genomically undercharacterized. Notably, while mitochondrial genome sequences have been documented for multiple congeners, a conspicuous genomic void persists for *R. czekanowskii* in public repositories.

This investigation provides the first high-resolution assembly of the complete mitochondrial genome for *R. czekanowskii*, bridging this critical taxonomic gap. Our findings establish essential molecular baselines for advancing phylogenetic reconstruction, resolving cryptic biodiversity patterns, and informing evidence-based conservation strategies for cold-adapted ichthyofauna in rapidly changing Northeast Asian watersheds.

## 2. Materials and methods

### 2.1. Sample collection and identification

**Figure 1.**
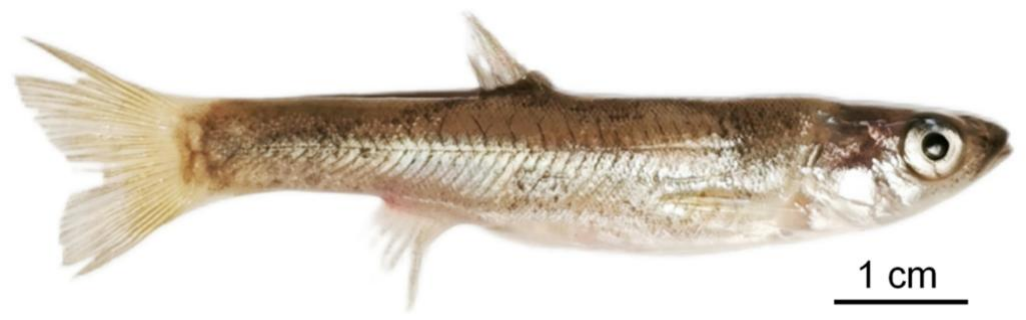
Reference image of *Rhynchocypris czekanowskii*. This photograph was taken by the author of this article, Yin-Tao Zhang.

During winter 2024, live specimens of *R. czekanowskii* were captured from Songhua River, Jiamusi City, China (46°52′02.99″ N, 130°27′28.19″ E). Species confirmation was achieved through standardized morphological analysis following the taxonomic framework (Freyhof and Kottelat 2007). Specimens were examined for key traits, including an incomplete lateral line that ends behind the pectoral tip, body depth ≤ 23 % SL, a long pointed male genital papilla, seven branched dorsal-fin rays and 33–36 mid-lateral scales. Specimens were documented photographically using a Nikon D850 camera prior to humane euthanasia *via* eugenol immersion (500 ppm concentration). Postmortem procedures included extraction of dorsal musculature using sterile dissection instruments, with tissue aliquots immediately cryopreserved in anhydrous ethyl alcohol (Sigma-Aldrich, ≥99.8% purity). Complete specimens underwent ethanol fixation (95% solution) and permanent archival deposition in the Ecological Specimen Repository at Lishui University (Accession code: LSU-2024-12-0024), with Jie Chen (jchen@lsu.edu.cn) serving as the contact person (Figure 1).

### 2.2. Mitochondrial genome assembly and annotation

Total genomic DNA was isolated from muscle specimens employing the Rapid Animal Genomic DNA Isolation Kit (Sangon Biotech, China). High-throughput sequencing was conducted on the Illumina HiSeq 2500 platform (150 bp paired-end mode), yielding 11.70 Gb of raw sequencing data. The raw reads were quality-filtered with Fastp v0.20.0 (Chen et al. 2018), resulting in 83,139,268 high-quality paired-end reads (10.37 Gb clean data). Clean reads were mapped to the mitochondrial genome of *Rhynchocypris jouyi* (GenBank accession number AB626852) using BWA-MEM v0.7.17 (Li 2013), and reads aligning to the reference were extracted for mitogenome-specific assembly. These reads were reassembled *de novo* using SPAdes v4.10 (Prjibelski et al. 2020) with k-mer sizes 21, 33, and 55. Gene annotation was conducted using MitoZ v2.4 (Meng et al. 2019) and MITOS WebServer (Matthias et al. 2013), with consistency checks. The circular mitochondrial genome map was visualized with Proksee (Grant et al. 2023). The coverage depth of the genome was determined using Bowtie2 v 2.3.4 (Langmead and Salzberg 2012) and SAMtools v1.16.1 (Li et al. 2009), and the sequencing depth and coverage map was drawn using ggplot2 (Ito and Murphy 2013) in R (Figure S1).

### 2.3. Phylogenetic analysis

In our phylogenetic analysis, we utilized previously published mitochondrial genomes from seven species of the genus *Rhynchocypris*, a newly obtained sequence from *R. titteya*, and seven species from other Cyprinid fish. *Danio rerio* from the subfamily Danioninae was the outgroup. The 13 PCG sequences were initially processed through PhyloSuite v1.2.1 (Zhang et al. 2020) for sequence extraction, followed by multiple sequence alignment performed in MAFFT v7.388 (Katoh and Standley 2013). Phylogenetic analysis was conducted based on maximum likelihood (ML) analyses implemented in IQ-TREE v2.1.2 (Minh et al. 2020) with the GTR + F + I + G4 nucleotide substitution model selected by ModelFinder (Kalyaanamoorthy et al. 2017). Support for the inferred ML tree was inferred by bootstrapping with 1,000 replicates. Phylogenetic trees were visualized and annotated using the Interactive Tree of Life (ITOL) (Letunic and Bork 2021).

## 3. Results

### 3.1. Mitochondrial genome characteristics

**Figure 2.**
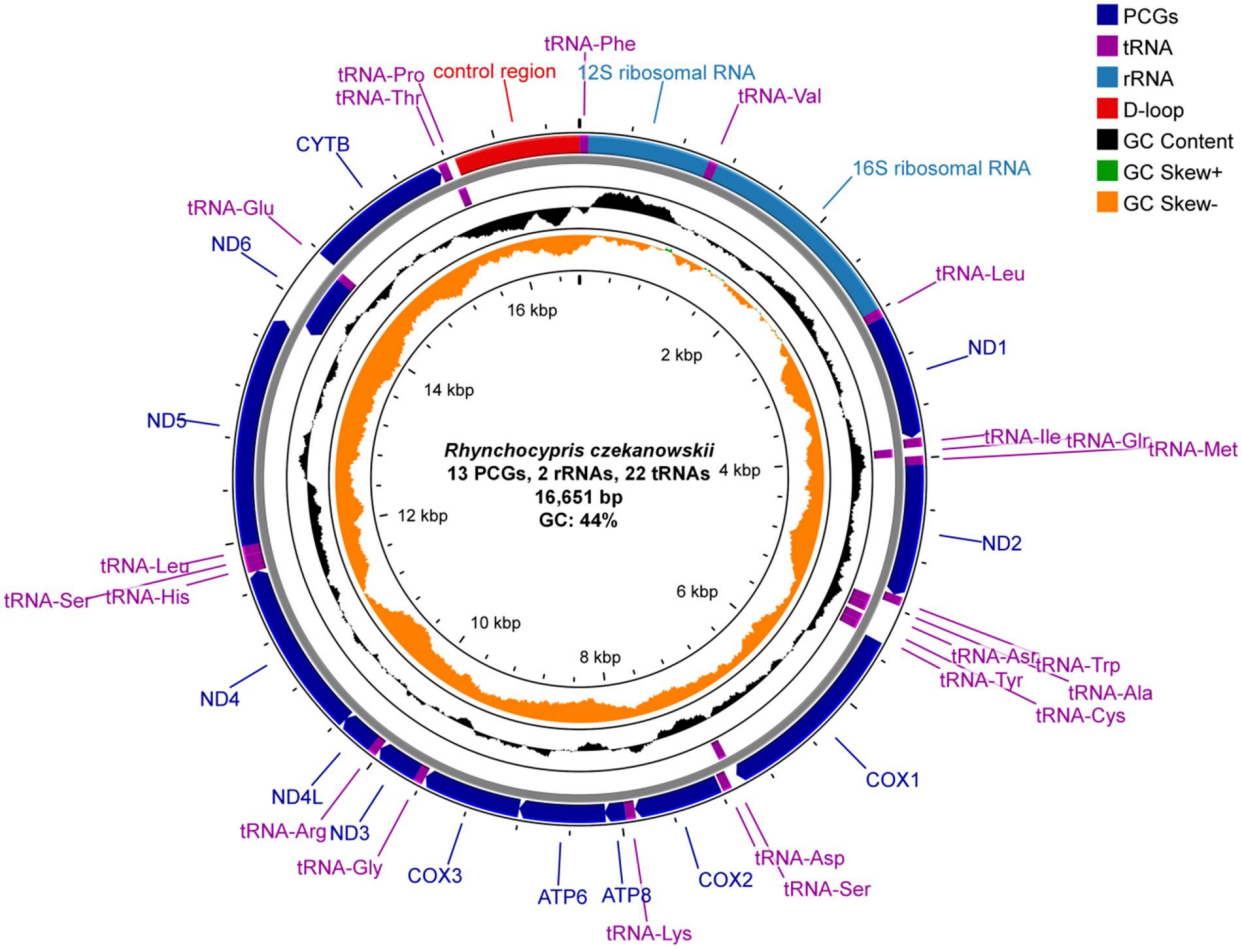
Circular map of the *Rhynchocypris czekanowskii* mitochondrial genome. The mitogenome is 16,651 bp long and contains 13 protein-coding genes, two rRNA genes and 22 tRNA genes. Arrows indicate transcriptional directions. Gene types and GC content/skew are color-coded.

The *R. czekanowskii* mitochondrial genome spans 16,651 bp, comprising 13 protein-coding genes (PCGs), 22 tRNA genes, 2 rRNA genes, and a control region. Nucleotide composition analysis revealed A: 28.65%, T: 27.34%, G: 17.65%, C: 26.35% (GC: 44%). Gene distribution exhibited strand asymmetry: the heavy strand encoded 28 genes (12 PCGs, 14 tRNA genes, and 2 rRNA genes) versus 9 genes (1 PCG, 8 tRNA genes) on the light strand. Except for *cox1*, which uses a GTG start codon, all other PCGs initiated with ATG start codons and terminated with canonical TAA/TAG stop codons. Incomplete stop codons (single T) were observed in *cox2*, *cox3*, and *cytb*. Transfer RNAs displayed lengths between 67 and 75 bp, while ribosomal RNA genes *16S ribosomal RNA* and *12S ribosomal RNA* measured 1689 bp (GC:44.52%) and 956 bp (GC:50.31%), respectively. The 981 bp control region (GC:36.60%) was localized between tRNA-Phe and tRNA-Pro (Figure 2).

### 3.2. Phylogenetic analysis

**Figure 3.**
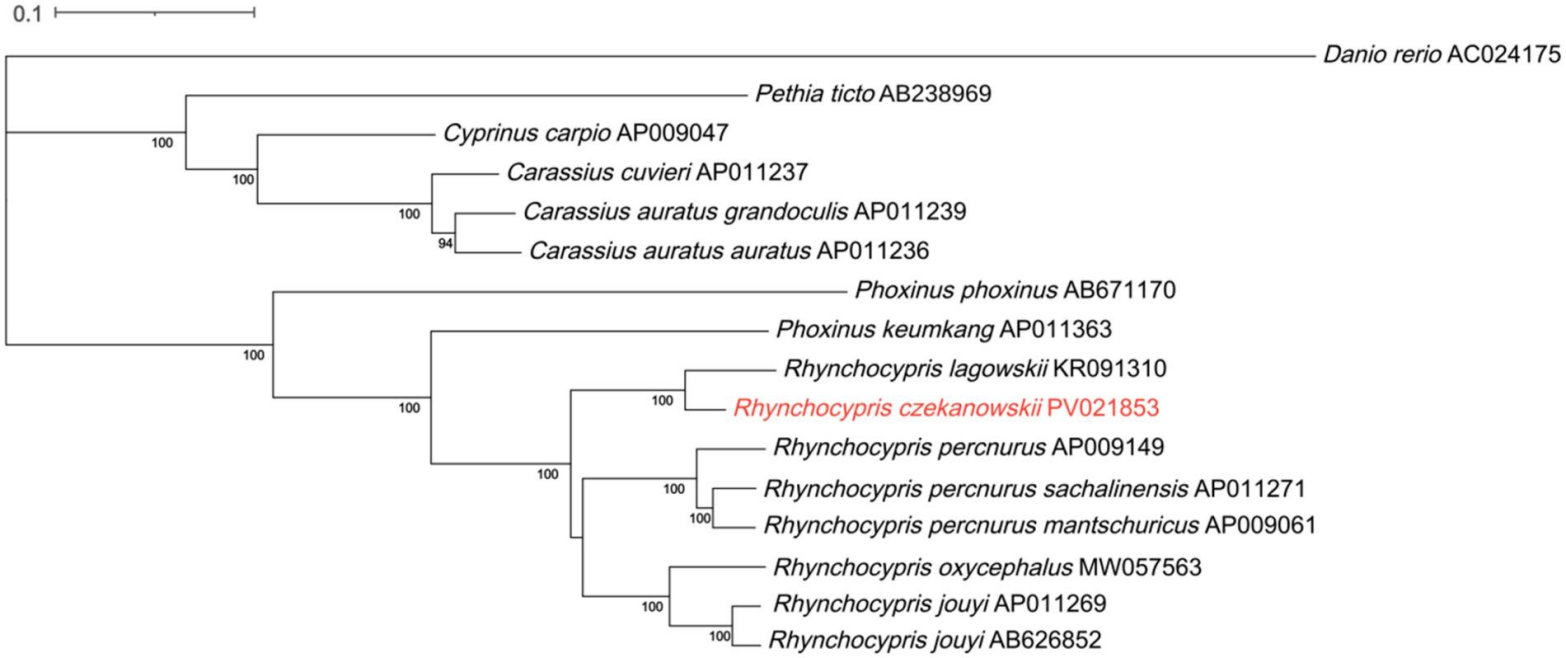
Maximum likelihood (ML) tree based on 16 mitogenome sequences of representative fish in Cyprinidae as the ingroup and *Danio rerio* as the outgroup. Numbers on the nodes are bootstrap values based on 1,000 replicates. The *Rhynchocypris czekanowskii* genome is marked in red font. Bootstrap values > 70% are displayed above the branches. The following sequences were used: *R. czekanowskii* PV021853 (this study), *Rhynchocypris jouyi* AB626852 (Imoto et al. 2013), *Rhynchocypris jouyi* AP011269 (Miya et al. 2015), *Rhynchocypris oxycephalus* MW057563 (Zhang et al. 2021), *Rhynchocypris lagowskii* KR091310 (unpublished), *Rhynchocypris percnurus* AP009149 (Imoto et al. 2013), *Rhynchocypris percnurus sachalinensis* AP011271 (Miya et al. 2015), *Rhynchocypris perenurus mantschuricus* AP009061 (Saitoh et al. 2006), *Phoxinus keumkang* AP011363 (unpublished), *Phoxinus phoxinus* AB671170 (Imoto et al. 2013), *Carassius auratus grandoculis* AP011239 (Miya et al. 2015), *Carassius auratus auratus* AP011236 (unpublished), *Carassius cuvieri* AP011237 (Miya et al. 2015), *Cyprinus carpio* AP009047 (Mabuchi et al. 2006), *Pethia ticto* AB238969 (Saitoh et al. 2006), and *Danio rerio* AC024175 (unpublished).

Maximum likelihood phylogenetic reconstruction based on mitochondrial genome sequences from eight *Rhynchocypris* species and eight representative Cyprinidae species strongly supported (100% bootstrap) the sister relationship between *R. czekanowskii* and *R. lagowskii*. These two species formed a well-defined monophyletic cluster within the *Rhynchocypris* genus, which showed clear evolutionary divergence from other Cyprinidae lineages in the reconstructed phylogeny (Figure 3). The evolutionary distance between species is represented by branch lengths in the tree, where shorter branches between closely related species, such as *R. czekanowskii* and *R. lagowskii*, suggest a small evolutionary distance (less than 0.05 nucleotide substitutions per site). The scale bar at the top of the figure indicates 0.1 nucleotide substitutions per site, and bootstrap support values above 70% indicate strong support for the branching relationships.

## 4. Discussion and conclusion

The present study delivers the first complete mitochondrial genome assembly for *R. czekanowskii*, addressing a critical taxonomic void in genomic resources for Northeast Asian freshwater ichthyofauna. Our comprehensive analysis reveals that the *R. czekanowskii* mitogenome (16,651 bp) maintains the conserved architecture characteristic of cyprinid mitochondrial DNA, while exhibiting lineage-specific variations with significant evolutionary implications. The observed A-T bias (55.99%) and structural conservation demonstrate remarkable congruence with congeneric species (*R. kumgangensis*: 54.06%; *R. oxycephalus*: 55.85%) (Yun et al. 2012; Zhang et al. 2021), suggesting strong purifying selection acting on mitochondrial genome organization within this genus. Selection is crucial for conserving mitochondrial genomes, with some regions under strong purifying selection to preserve functions like ATP production and oxidative phosphorylation, making them more conserved across species. However, demographic factors such as population size, genetic drift, and bottlenecks can affect mitogenomic diversity. In small or isolated populations, genetic drift may decrease mitochondrial diversity, causing the loss of variants that selection might retain in larger, stable populations.

Phylogenetic reconstruction employing maximum-likelihood methods robustly resolves *R. czekanowskii* as the sister species to *R. lagowskii* (BS = 100%). This phylogenetic proximity receives compelling support from molecular evidence: *R. lagowskii* not only shares the ATP haplotype with *R. czekanowskii*, but also possesses the most closely related Cytb haplotype within currently characterized phylogroups (Kusznierz et al. 2023).

Two genomic peculiarities warrant special emphasis: First, the GTG initiation codon in *cox1*, while atypical for vertebrates, appears conserved across multiple cyprinid lineages (Islam et al. 2020; Lee et al. 2023; Wang et al. 2023), potentially representing a family-level synapomorphic characteristic. Second, the identification of truncated stop codons (single T) in *cox2*, *cox3*, and *cytb* suggests reliance on post-transcriptional polyadenylation for termination signal completion - a mechanism well-documented in teleost mitogenomes (Wang et al. 2013; Guo et al. 2016; Szafranski 2017; Yang et al. 2019). These molecular idiosyncrasies highlight the necessity for standardized comparative annotation protocols in mitochondrial genome analyses.

## Supplementary Material

Figure S1.doc

## Data Availability

The genome sequence data supporting this study are openly available in GenBank of NCBI at https://www.ncbi.nlm.nih.gov under the accession number PV021853. The associated BioProject, SRA, and Biosample numbers are PRJNA1215793, SRR32129882, and SAMN46419660, respectively.
