# Supplementary material for: The complete mitochondrial genome of Rhynchocypris czekanowskii (Cypriniformes, Cyprinidae)
Source: Mitochondrial DNA B Resour. 2025 Jul 23;10(8):763–7. doi: 10.1080/23802359.2025.2535639 (PMC12288181; doi:10.1080/23802359.2025.2535639)
Supplement: Figure S1.doc [file TMDN_A_2535639_SM8249.doc]

**The complete mitochondrial genome of *Rhynchocypris czekanowskii* (Cypriniformes, Cyprinidae)**

Yin-Tao Zhang, Cheng-Pu Lu, Yu-Hui Tao, Cheng-Wei Tong, Jie Chen, Wei Liu

**Figure S1.** Depth of coverage for *Rhynchocypris czekanowskii* mitochondrial genome. X and Y axis present nucleotide position of *R. czekanowskii* mitochondrial genome and coverage depth, respectively.
